# Enhanced Path Travel Time Prediction via Guided Fusion of Heterogeneous Sensors Using Continuous-Time Dynamics

**DOI:** 10.3390/s25185873

**Published:** 2025-09-19

**Authors:** Ang Li, Hanqiang Qian, Yanyan Chen

**Affiliations:** Beijing Key Laboratory of Traffic Engineering, Beijing University of Technology, Beijing 100021, China; liang@bjut.edu.cn (A.L.); qianhq@emails.bjut.edu.cn (H.Q.)

**Keywords:** travel time prediction, heterogeneous sensor fusion, Neural Ordinary Differential Equations, intelligent transportation systems

## Abstract

Accurate path travel time prediction is often hindered by sparse and heterogeneous traffic data. This paper proposes FusionODE-TT, a novel model designed to address these challenges by modeling traffic as a continuous-time process. The model features a Recurrent Neural Network encoder that processes multi-source time-series data to initialize a latent state vector, which then evolves over the prediction horizon using a Neural Ordinary Differential Equation (NODE). The core innovation is a guided fusion mechanism that leverages sparse but high-fidelity Automatic Vehicle Identification (AVI) data to apply strong, event-based corrections to the model’s continuous latent state, mitigating error accumulation in the prediction process. Experiments were conducted on a real-world dataset comprising AVI, GPS, and point sensor data from a major urban expressway. The experimental results demonstrate that the proposed model achieves superior accuracy, outperforming a suite of baseline models in terms of prediction accuracy and robustness. Furthermore, a comprehensive ablation study was performed to validate the efficacy of our design. The study quantitatively confirms that both the continuous-time dynamics modeled by the NODE and the guided fusion mechanism are essential components, each providing a significant and independent contribution to the model’s overall performance.

## 1. Introduction

Urban transportation networks must be efficient, safe, and sustainable. Intelligent transportation systems (ITS) are critical for achieving these goals. At the heart of any effective ITS is accurate and reliable travel time prediction (TTP). TTP is a cornerstone capability. It supports many vital applications. These include dynamic route guidance for drivers and fleet operators [1]. They also include optimized traffic signal control and better incident management.

For travelers, precise TTP allows for smarter decisions. This reduces travel delays and enables more effective trip planning. For traffic authorities, TTP provides essential tools. They can use these tools to proactively manage congestion. This also helps lower fuel consumption and emissions. Ultimately, a reliable TTP system improves the entire network’s resilience [2]. Despite decades of research and deployment, achieving consistently high-fidelity TTP, particularly across complex urban and freeway networks and under dynamic conditions, remains a formidable scientific and engineering challenge [1]. This challenge stems fundamentally from the inherent limitations and complementary nature of the diverse sensor modalities currently employed for traffic data acquisition [3]. Traditionally, ITS architectures have relied heavily on three primary data streams.

Technologies such as inductive loop sensors, radar, and microwave sensors provide high-frequency measurements of traffic variables (e.g., volume, occupancy, spot speed) at fixed locations. While offering valuable temporal granularity, their spatial resolution is inherently limited [4]. Estimating segment or path travel times from these point measurements necessitates spatial extrapolation, a process fraught with inaccuracies, particularly under non-homogeneous traffic flow conditions often prevalent during congestion onset or dissipation. Furthermore, single-loop sensors, the most widely deployed variant, do not directly measure speed, requiring inference based on occupancy and assumed vehicle lengths, introducing further uncertainty. Sensor malfunctions, calibration drift, and counting errors also contribute to data quality issues [5].

Systems like Automatic Vehicle Identification (AVI), License Plate Recognition (LPR), Bluetooth/Wi-Fi MAC address matching, and Dedicated Short-Range Communications (DSRC) offer direct measurements of segment travel times by re-identifying vehicles at two or more points along a corridor [6]. These measurements are generally considered highly accurate representations of the experienced travel time for the sampled vehicles. However, the deployment infrastructure for such systems is often sparse, both spatially (covering limited network segments) and temporally (yielding infrequent updates, especially during low-flow periods). A critical limitation is the inherent latency; these systems typically report Arrival-based Travel Time (ATT), reflecting conditions experienced by vehicles that have completed their journey through the segment. This ATT can significantly diverge from the real-time Predicted Travel Time (PTT), especially when traffic conditions are rapidly evolving [7].

Leveraging Global Positioning System (GPS) or other localization technologies embedded in smartphones, fleet vehicles (taxis, trucks, delivery vans), or dedicated probe vehicles provides trajectory data, offering the potential for broader network coverage compared to fixed infrastructure. Floating Car Data derived from these probes can yield direct or inferred travel time estimates. However, practical limitations persist. Market penetration rates are often insufficient to guarantee dense spatiotemporal coverage, leading to data sparsity [8]. GPS measurements themselves are subject to noise, signal obstructions, and drift. Furthermore, accurately associating GPS traces with specific road segments requires sophisticated map-matching algorithms, which can introduce additional errors and computational overhead [9].

The individual shortcomings of these sensor modalities underscore the compelling need for advanced data fusion techniques [10]. Data fusion is more accurate, reliable, comprehensive, and robust than could be obtained from any single source alone. The fundamental premise is to leverage the complementary strengths. For instance, the high accuracy of sparse AVI data, the high temporal frequency of potentially noisy point sensor data, and the broader spatial sampling (albeit potentially sparse and noisy) of GPS probes can construct a superior representation of the underlying traffic dynamics. The core challenge lies not merely in aggregating data, but in intelligently integrating streams possessing vastly different characteristics in terms of accuracy, precision, spatial and temporal resolution, latency, and noise profiles [11].

Existing data fusion methodologies for TTP span a spectrum of approaches. Classical statistical and filtering techniques have been widely explored. Weighted averaging methods offer simplicity but struggle to optimally combine sources with disparate quality or conflicting information [12]. Bayesian inference provides a probabilistic framework for evidence combination but often requires strong prior assumptions [13]. Dempster-Shafer (D-S) evidence theory offers a mechanism to handle uncertainty and conflict without priors. However, it can be computationally complex and sensitive to the definition of belief structures, particularly when dealing with continuous variables like travel time [10]. Kalman Filtering (KF) and its non-linear extensions, the Extended KF (EKF) [14] and the Unscented KF (UKF) [15], represent a powerful paradigm for state estimation in dynamic systems, explicitly modeling process and measurement noise. However, the performance of KF methods hinges critically on the accuracy of the underlying system dynamic model and the appropriateness of Gaussian noise assumptions.

In parallel, the advent of deep learning (DL) has ushered in powerful data-driven paradigms for TTP and fusion. Models such as Artificial Neural Networks, and particularly sequence models like Long Short-Term Memory (LSTM) and Gated Recurrent Units (GRU) have demonstrated remarkable capabilities in capturing complex, non-linear temporal dependencies in traffic data [16]. More recently, Graph Neural Networks (GNNs) have emerged as the state-of-the-art for modeling the explicit network topology and spatial dependencies inherent in traffic systems [17]. Transformer architectures, leveraging self-attention mechanisms, excel at capturing long-range dependencies and are increasingly applied to TTP [18]. While DL models offer unparalleled flexibility and performance, they often function as black boxes and lack interpretability. They typically require vast amounts of labeled training data, and their generalization performance beyond the training distribution can be uncertain.

Despite these advancements, a critical gap remains in the principled fusion of highly heterogeneous data streams characterized by significant disparities in precision and sparsity, particularly within a continuous-time modeling framework. Existing methods often treat all data sources somewhat uniformly within their respective frameworks [10] (e.g., assigning different noise variances in KF or relying on the model to implicitly learn weights in DL) but lack an explicit mechanism to leverage sparse, high-precision measurements (like AVI) for strong, corrective updates to the system state, while simultaneously using denser, albeit potentially less precise or indirect, measurements (like GPS trajectories or loop sensor readings) for continuous guidance of the state evolution between these corrections. Simple averaging or standard filtering/learning approaches risk either diluting the high-value information from sparse sensors or becoming overly reliant on potentially biased, high-frequency data. Effectively harnessing the unique value proposition of each data type demands a more nuanced fusion strategy.

Furthermore, traffic dynamics are inherently continuous processes evolving over time. While discrete-time models like Recurrent Neural Networks (RNNs) approximate these dynamics, they can struggle with irregularly sampled data (common with GPS and AVI) and may not fully capture the underlying continuous evolution [8,19]. Neural Ordinary Differential Equations (NODEs) and their extensions, such as Neural Controlled Differential Equations (NCDEs), have recently emerged as a powerful class of deep learning models. They learn system dynamics directly in continuous time by parameterizing the derivative of the hidden state with a neural network. This continuous-time formulation makes them naturally suited for handling irregular time series data and modeling underlying physical processes. However, challenges remain regarding their computational cost (requiring ODE solvers), training stability, and inherent difficulty in modeling abrupt, discontinuous events (like traffic incidents or sudden congestion shifts) which are not well-described by standard ODEs.

To address the aforementioned limitations, this paper introduces FusionODE-TT, a novel model for accurate TTP based on the principled fusion of heterogeneous traffic sensor data within a continuous-time modeling paradigm. The core innovation of FusionODE-TT lies in its guided fusion mechanism, implemented within a NODE architecture. This mechanism explicitly differentiates between data sources based on their characteristics.

First, sparse but high-precision AVI-derived travel time measurements, when available, are used to apply a strong corrective update to the latent system state H(t), effectively anchoring the model estimate to ground-truth observations. This concept draws inspiration from the update step in Kalman filtering and data assimilation techniques used in fields like weather forecasting, where sparse, high-impact observations are integrated. Second, denser but potentially lower-precision or indirect data streams from GPS trajectories and point sensors are utilized to provide continuous guidance to the state evolution process modeled by the NODE’s derivative function. This guidance helps the model infer state dynamics more accurately, particularly during periods lacking high-precision AVI updates.

Therefore, the contributions of this work are threefold:

First, we propose a novel guided fusion mechanism embedded within a NODE framework, specifically designed to differentially leverage heterogeneous traffic data for path travel time prediction.

Second, we detail this mechanism for integrating AVI, GPS, and point sensor data, and leverage sparse AVI data for strong correction of the latent state of path travel time.

Third, real-world datasets in Hong Kong have been used for validation of the proposed FusionODE-TT model, and the performance is satisfactory.

This paper is organized as follows: Section 2 reviews related work in data fusion and continuous-time traffic modeling in detail. Section 3 presents the proposed FusionODE-TT model, elaborating on the NODE backbone and the guided fusion mechanism. Section 4 describes the experimental setup and performance evaluation of the proposed FusionODE-TT model. Section 5 provides the discussion on the efficacy of continuous-time guided fusion, model interpretability, and limitations. Finally, Section 6 concludes the paper and outlines directions for future research.

## 2. Literature Review

The accurate TTP in dynamic transportation networks, leveraging data from heterogeneous sensor sources, resides at the confluence of several research domains, including traffic flow theory, statistical estimation, machine learning, and sensor fusion. This section provides a critical review of the pertinent literature, focusing on existing methodologies for fusing disparate traffic data streams for TTP.

### 2.1. Heterogeneous Data Fusion for Travel Time Prediction

The imperative for data fusion arises directly from the complementary limitations of prevalent traffic sensors [20]. Point sensors offer temporal richness but spatial poverty, interval sensors provide accurate segment times but suffer from spatiotemporal sparsity and latency, and probe vehicles yield broader potential coverage but face challenges of penetration rates, noise, and map-matching inaccuracies. Consequently, a significant body of research has focused on developing fusion algorithms to synergize these sources.

Methodologies for fusing heterogeneous data in traffic prediction can be broadly categorized. Statistical and filtering approaches represent a classical line of inquiry. For instance, El Faouzi et al. [21] demonstrated that Dempster-Shafer evidence theory can be used to fuse data from loop detectors and toll stations, generating an improved and more robust travel time estimate by formally combining belief values calculated from each source. More advanced filtering techniques, such as the Tensor Extended Kalman Filter (TEKF), have been proposed by Chang et al. [14] to overcome the limitations of the standard EKF, which is restricted to vector-form state variables. Their work shows the feasibility of handling multi-relational traffic data in its natural tensor form while still capturing non-linear dynamics.

In recent years, machine learning (ML) and deep learning (DL) approaches have dominated, treating fusion as a learning problem. Bing et al. [22] developed a hybrid model combining k-Nearest Neighbors (k-NN) with a Least Squares Support Vector Regression (LSSVR), finding that this combination yielded higher estimation accuracy for arterial roads than other baseline methods. Deep learning has further advanced the state-of-the-art. Bogaerts et al. [23] showed that a Graph CNN-LSTM architecture can effectively extract spatio-temporal features from sparse GPS data, outperforming standard LSTM models for both short and long-term predictions up to 4 h. To further improve upon GNNs, Hu et al. [18] introduced a Graph Transformer network that constructs multiple graphs to model the complex network structure, achieving an average improvement of 9.78% in mean absolute error (MAE) over existing methods on benchmark datasets. The use of attention mechanisms, as demonstrated by Abdelraouf et al. [24], not only improves performance by learning to weigh the contributions of short-term, daily, and weekly traffic patterns but also provides valuable interpretability by highlighting the importance of these periodic inputs.

Despite this progress, effectively fusing the specific triad of AVI, GPS, and point sensor data, considering their unique signatures of accuracy, sparsity, latency, and noise, remains an open challenge requiring tailored strategies beyond generic fusion frameworks [20]. Moreover, a significant drawback of recent data-driven fusion methods is that they often function as “black boxes,” posing challenges for the interpretability of their internal states and decision-making processes.

### 2.2. Handling Sparse, High-Precision Data

AVI and similar interval-based measurements present a specific challenge. They offer high-fidelity ground truth but are often sparse in time and space. Naive fusion risks diluting this valuable information. Several strategies aim to address this. Weighted fusion explicitly assigns higher importance to high-precision sources, often based on inverse variance, sample size, or entropy-based quality metrics. Interpolation and extrapolation techniques are used to fill gaps, ranging from simple methods to more sophisticated spatial (e.g., Kriging) or temporal approaches (e.g., using historical patterns or other sensors) [25]. However, simple interpolation struggles with complex dynamics, and extrapolation remains inherently difficult, especially with high sparsity. Recent work explores spatiotemporal reconstruction for sparse, unstructured data.

Attention mechanisms within DL models can implicitly learn to focus on high-value inputs when available. Sparse attention variants aim to improve efficiency in such scenarios. Perhaps the most principled framework for integrating sparse observations is Data Assimilation (DA), widely used in fields like meteorology. DA techniques, including KF and its variants (EKF, UKF, EKF), systematically combine model predictions with observations. The Kalman gain inherently weights observations based on their uncertainty relative to the model’s prediction uncertainty [15]. High-precision AVI data naturally leads to a larger gain and thus a stronger correction of the predicted state.

While these approaches are effective, they typically treat the sparse, high-precision data as just another observation to be assimilated or as an input to be weighted [14,15]. A clear research gap exists for frameworks that can more explicitly leverage these sparse, high-fidelity measurements as a direct “guidance” or “strong correction” signal to recalibrate the model’s internal state, particularly when the model is simultaneously processing other, more noisy, high-frequency data streams.

### 2.3. Continuous-Time Modeling with NODEs/NCDEs

Recognizing that traffic dynamics are fundamentally continuous, researchers have begun exploring Neural Differential Equations (NDEs) as an alternative to traditional discrete-time models. NODEs model the hidden state evolution using a neural network. NCDEs extend this by allowing the dynamics to be driven by an external control path, making them particularly suitable for time series influenced by exogenous inputs.

NDEs have been applied to various transportation problems. First, it has been applied in traffic flow prediction [26,27,28]. Second, regarding trajectory modeling, NDEs can model the continuous movement of vehicles [29] or pedestrians [30]. Hence, the corresponding human mobility can be predicted. Third, compared to discrete-time models like RNNs, NDEs offer a more natural representation of continuous dynamics and superior handling of irregular sampling. Thus, handling irregular data can be achieved by NDEs, as the ODE solver can integrate between any two time points [31].

While these NDEs provide an elegant framework for continuous-time modeling, a key limitation is that once the dynamic function is learned, the system evolves autonomously from an initial condition [26,28]. They lack a formal mechanism to incorporate and be corrected by discrete, high-precision observations that may arrive during the prediction horizon. This can lead to trajectory drift, where the model’s predicted state diverges from reality over time. Therefore, a significant methodological gap exists in developing a continuous-discrete framework: one that combines the continuous state evolution of NDEs with a principled, event-based correction mechanism inspired by data assimilation. Such a framework could prevent trajectory drift by re-anchoring the model’s state to high-quality observations, a challenge that this paper aims to address.

### 2.4. Summary and Identified Gaps

Previous sub-sections reveal several persistent gaps that the proposed model, FusionODE-TT, is designed to address.

First, as established in our review of data fusion techniques (Section 2.1), a persistent research gap exists in developing fusion frameworks specifically tailored to the unique and often conflicting characteristics of the AVI, GPS, and point sensor triad. Moreover, a long-standing challenge is the trade-off between the predictive power of complex models and their lack of interpretability. This paper’s contribution is to propose a novel hybrid framework that provides high accuracy while maintaining a degree of interpretability and trustworthiness.

Second, as highlighted in the discussion on handling sparse data (Section 2.2), while various methods exist, there is a need for frameworks that can more explicitly leverage sparse, high-precision data as a corrective signal to the model’s internal state. This paper details a specific mechanism for integrating AVI, GPS, and point sensor data, and leverages sparse AVI data for strong correction of the latent state of path travel time.

Finally, as noted in the review of continuous-time modeling (Section 2.3), a methodological gap exists in developing models that can embed the event-based correction principles of data assimilation directly within a continuous-time deep learning model. This paper proposes a novel guided fusion mechanism embedded within a NODE framework, specifically designed to differentially leverage heterogeneous traffic data for path travel time prediction.

In conclusion, the contributions of this paper, which include a new model, a detailed strong correction mechanism, and the validation of its performance on real-world datasets, are designed to holistically address these identified gaps.

## 3. Methodology

This section delineates the methodological foundation of the FusionODE-TT model, conceived for high-fidelity TTP through the principled fusion of heterogeneous traffic sensor data. Our approach leverages the expressive power of continuous-time dynamics modeling, instantiated via NODEs, coupled with a novel guided fusion mechanism. This mechanism is specifically architected to synergistically integrate high-frequency, potentially noisy guidance data derived from point sensors and probe vehicles, with sparse, yet high-accuracy, corrective data obtained from interval (AVI-type) sensors. We first establish a formal problem definition and introduce the notation employed throughout this section. Subsequently, we detail the NODE-based core architecture, followed by an in-depth exposition of the guided fusion components—continuous guidance and strong correction. Finally, we discuss the model training procedure and pertinent implementation considerations.

### 3.1. Problem Statement

The overarching goal of this work is to develop a robust predictive model capable of accurately forecasting the travel time along a specified route within a complex transportation network, utilizing a diverse array of sensor inputs available in contemporary ITS.

**Network Representation:** We model the transportation infrastructure as a directed graph $G=\left(V,E\right)$, where V represents the set of nodes (e.g., intersections, significant junctions, sensor locations) and E denotes the set of directed edges, corresponding to unidirectional road segments connecting pairs of nodes. A path or route p through the network is defined as a temporally ordered sequence of k contiguous segments, $p=\left(s_{1},s_{2},\ldots ,s_{k}\right)$, where each segment $s_{k}\in E$.

**Heterogeneous Data Sources:** The FusionODE-TT model is designed to ingest and process data from three principal sensor modalities, each possessing distinct characteristics.

**Point Sensors:** These sensors (e.g., inductive loops, radar, microwave sensors) are deployed at fixed locations l, typically associated with a specific road segment s∈E. They provide time-stamped measurements of spot speed. The point sensor dataset ($D_{L}$) comprises tuples of the form: $D_{L}=\left(l_{i},t_{i},v_{i}\right)$. $l_{i}$ gives the location of sensor i, $t_{i}$ is the measurement timestamp, $v_{i}$ denotes the measured spot speed. Data from $D_{L}$ is characterized by high temporal resolution but provides only point-specific information, necessitating spatial inference to estimate segment-level conditions. Furthermore, this data is susceptible to noise and sensor malfunctions.

**Interval Sensors:** Systems such as AVI using toll tags, License Plate Recognition (LPR), or Bluetooth/Wi-Fi re-identification provide direct measurements of the time taken by individual vehicles to traverse a specific path p. The dataset, $D_{A}$, contains records like: $D_{A}=\left(\;{veh}_{m,p},t_{m,p}^{entry},t_{m,p}^{exit}\right)$,where ${veh}_{m,p}$ identifies the vehicle m traversing path p, $t_{m,p}^{entry}$ is the timestamp recorded upon entering path p, and $t_{m,p}^{exit}$ is the timestamp upon exiting. The directly observed travel time for this traversal, often referred to as Arrival-based Travel Time (ATT), is calculated as $\tau_{m,p}=t_{m,p}^{exit}-t_{m,p}^{entry}$. Data from $D_{A}$ is generally considered the most accurate ground-truth measurement of experienced travel time for the sampled vehicles. However, its primary limitations are significant spatiotemporal sparsity (due to limited deployment or low detection rates) and inherent latency, as the travel time $\tau_{m,p}$ only becomes known at $t_{m,p}^{exit}$, reflecting conditions experienced in the past time intervals.

**Probe Vehicles:** This data stream originates from vehicles equipped with localization devices, primarily GPS. The dataset, $D_{G}$, consists of time-stamped trajectory points for individual vehicles: $D_{G}=\left({veh}_{j},\;t_{j},{lat}_{j},{lon}_{j},v_{j}\right)$, where ${veh}_{j}$ is a unique vehicle identifier, $t_{j}$ is the timestamp, ${lat}_{j}$ and ${lon}_{j}\;$ are the latitude and longitude of vehicle j, and $v_{j}$ is the instantaneous speed of vehicle j. Probe vehicles data offers the potential for extensive network coverage, overcoming the fixed-location limitation of point sensors. However, its practical utility is often constrained by variable and sometimes low market penetration rates (leading to spatiotemporal sparsity), inherent GPS positional noise and inaccuracies (especially in challenging environments like urban canyons), and the critical dependence on accurate map-matching algorithms to associate trajectory points with the network graph G.

**Prediction Objective:** The central aim is to predict the PTT for a given path p, denoted $\tau_{p}\left(t,∆t\right)$. This represents the anticipated duration required for a hypothetical vehicle, commencing its journey along path p at time t, to reach the end of the path. The prediction horizon ∆t specifies the future time window for which the prediction is relevant (e.g., predicting the travel time for the next 30 min). The PTT inherently requires forecasting the evolution of traffic conditions along the path p during the travel period itself. It distinguishes fundamentally from the lagged ATT measurements provided by interval sensors.

### 3.2. The Proposed Model

**Figure 1:**
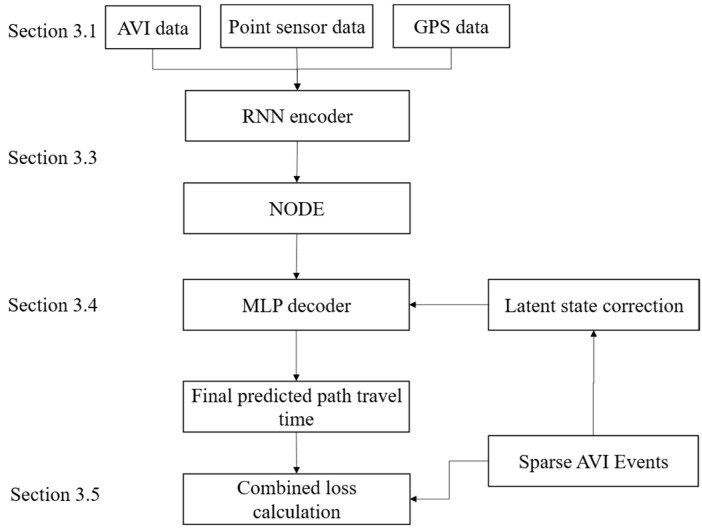
The proposed FusionODE-TT model.

To address the challenges of path travel time prediction using sparse and heterogeneous data stated in Section 3.1, we propose a novel model, FusionODE-TT, which is designed to effectively model the underlying traffic dynamics in continuous time. The architecture of this framework, as illustrated in Figure 1, is systematically composed of three primary modules: an encoder for latent state initialization, a core dynamic modeling block based on NODEs, and a decoder for the final prediction task.

First, time-aligned feature sequences derived from heterogeneous sensor data and historical travel times are fed into an RNN encoder (Section 3.3). This module processes the input sequence to generate a fixed-dimensional latent state vector, $H\left(t_{0}\right)$, which encapsulates the comprehensive traffic state of the path at the beginning of the prediction horizon. This vector serves as the initial condition for the core dynamic modeling block.

The core of our framework is the guided continuous-time dynamics module (Section 3.3 and Section 3.4), which models the evolution of the traffic state $H\left(t\right)$ using a NODE. This approach represents the traffic flow as a continuous process, offering a more physically plausible model than traditional discrete-time methods. A key innovation within this module is the guided fusion mechanism. This mechanism leverages sparse but highly accurate travel time observations from AVI sensors to correct the ODE’s latent state trajectory. When an AVI event is observed, the model simulates the travel time, calculates the error against the observation, and uses a gain network to compute a corrective term that recalibrates the latent state. This process anchors the model’s internal state to real-world observations, significantly enhancing long-term prediction accuracy.

The final component of our proposed framework is the decoder, which is responsible for mapping the terminal latent state vector, $H\left(T\right)$, into the final path travel time prediction. The latent state $H\left(T\right)$ is the output of the core module, representing the culmination of the system’s continuous-time evolution, guided by the fusion mechanism. For this decoding task, we employ a standard Multi-Layer Perceptron (MLP). The MLP is a powerful yet efficient function approximator, well-suited for this regression task. It takes the high-dimensional latent state $H\left(T\right)$ as input and processes it through a series of non-linear transformations (i.e., hidden layers with ReLU activation functions) to produce a single, scalar output value. This output represents the predicted travel time in minutes. The entire FusionODE-TT model, including the encoder, the Neural ODE, and the MLP decoder, is trained end-to-end. This is achieved by minimizing a composite loss function.

### 3.3. Continuous-Time Dynamics with NODEs

The initial step in our framework is to process the time-aligned, multivariate input sequence derived from the various sensors and historical records. This input, representing the traffic state over a historical lookback window, is fed into an RNN encoder. The primary function of this encoder is to learn and compress the complex temporal dependencies within the input sequence into a fixed-dimensional hidden state vector. This vector is then transformed through a linear projection layer to produce the initial latent state, denoted as $H\left(t_{0}\right)$. This latent state serves as a comprehensive summary of the path’s traffic condition at the beginning of the prediction horizon and provides the initial value for the subsequent continuous-time dynamic modeling block.

The FusionODE-TT model adopts a continuous-time perspective on traffic dynamics, diverging from traditional discrete-time modeling approaches. We hypothesize that the complex, evolving state of the traffic network can be effectively captured by a lower-dimensional latent state vector $H\left(t\right)\in R^{d}$, where d is the dimensionality of this latent representation. $H\left(t\right)$ is intended to implicitly encode all pertinent information about traffic conditions (e.g., speeds, densities, queue lengths) across the network segments relevant to the prediction task at time t.

The temporal evolution of this latent state is modeled using a NODE, where the core dynamics function, f, is parameterized by a neural network with learnable parameters θ. A foundational challenge with any purely self-evolving dynamic system is its potential to drift from the true state over time if it is not continuously anchored to real-world observations. The significance of introducing continuous guidance signals, ($X_{L}\left(t\right),X_{G}\left(t\right)$), is to directly address this challenge. These signals, which are derived from our heterogeneous sensor data, provide the ODE with a continuous stream of external, real-time information about the traffic environment. By conditioning the latent state’s evolution on these signals—in addition to the road network’s spatial structure, represented by the graph $G=\left(V,E\right)$—we ensure the model’s dynamics are constantly informed by and responsive to real-time traffic conditions, rather than evolving in isolation. This leads to the governing equation of our framework.

The temporal evolution of this latent state is defined as:

(1) $$\frac{dH\left(t\right)}{dt}=f\left(H\left(t\right),X_{L}\left(t\right),X_{G}\left(t\right),t;\theta \right)$$

To effectively model the spatiotemporal dynamics, the function f internally leverages a Graph Neural Network (GNN). At each evaluation step within the ODE solver, f first uses a GNN layer to update the state based on neighbors in $G=\left(V,E\right)$ (with adjacency matrix A), capturing spatial interactions:

(2) $$H_{\mathrm{spatial}}\left(t\right)=\mathrm{GNN}\left(H\left(t\right),A;\theta_{\mathrm{gnn}}\right)$$

Then, it combines this spatially aware state $H_{\mathrm{spatial}}\left(t\right)$ with the original state $H\left(t\right)$ and the continuous guidance inputs $X_{L}\left(t\right)$, $X_{G}\left(t\right)$ using a temporal processing block (e.g., an MLP) to compute the final derivative, which represents the output of f:

(3) $$f\left(\ldots \right)=\mathrm{MLP}\left(H_{\mathrm{spatial}}\left(t\right),H\left(t\right),X_{L}\left(t\right),X_{G}\left(t\right),t;\theta_{\mathrm{mlp}}\right)$$

The state $H\left(t\right)$ at any time t, given an initial state $H\left(t_{0}\right)$, is determined by the solution to the initial value problem defined by Equation (1), obtained via integration:

(4) $$H\left(t\right)=H\left(t_{0}\right)+\int_{t_{0}}^{t}f\left(H\left(s\right),X_{L}\left(s\right),X_{G}\left(s\right),s;\theta \right)ds$$

The continuous nature of Equation (4) provides inherent advantages over discrete-time models. It naturally handles irregularly sampled input data without requiring explicit imputation or awkward padding schemes, and it can, in principle, model the underlying continuous physical processes more faithfully.

To translate the learned latent state $H\left(t\right)$ into the desired output, which is the predicted travel time, a decoder function g is implemented as a neural network with learnable parameters ϕ.

(5) $${\hat{\tau }}_{p}\left(t,\Delta t\right)=g\left(H\left(t\right);\phi \right)$$

where the decoder maps the latent state at the prediction initiation time t to the PTT estimate ${\hat{\tau }}_{p}\left(t,\Delta t\right)$ for the specified path p. The prediction horizon Δt might influence the training target or could be an additional input to g.

### 3.4. The Guided Fusion Mechanism

A central innovation of FusionODE-TT is the guided fusion mechanism, designed to intelligently integrate the heterogeneous data streams ($D_{G}$, $D_{L}$, $D_{A}$) by explicitly recognizing and leveraging their distinct characteristics within the NODE framework. It comprises two complementary components: continuous guidance and strong correction, which are illustrated in Section 3.4.1 and Section 3.4.2, respectively.

#### 3.4.1. Continuous Guidance

Data from point sensors ($D_{L}$) and probe vehicles ($D_{G}$) typically arrive more frequently than AVI data, providing a continuous, albeit potentially noisy and incomplete, stream of information about traffic conditions. This stream is used to continuously “guide” the trajectory of the latent state $H\left(t\right)$ as it evolves according to the learned dynamics.

Raw data from $D_{L}$ and $D_{G}$ should be processed into feature vectors. For point sensors, ($t_{i},v_{i}$) from point sensor $l_{i}$ relevant to the path P are aggregated over short, regular time intervals (e.g., 2 min). This aggregation might involve averaging, median filtering, or more sophisticated methods. The resulting aggregated features for all relevant sensors at time interval j form a feature vector $X_{L}\left(t_{j}\right)$. GPS trajectory points are first subjected to map-matching to associate them with network segments $s_{k}\in E$. Once matched, segment-level statistics (e.g., average speed, vehicle density estimates based on probe counts and segment length) are computed by aggregating matched points within short time intervals, yielding feature vectors $X_{G}\left(t_{j}\right)$.

To integrate these discretely observed features into the continuous-time dynamics, we must define their continuous-time counterparts, $X_{L}\left(t\right)$ and $X_{G}\left(t\right)$. We employ piecewise-constant interpolation. Under this strategy, feature values remain constant between updates:

(6) $$X_{L}\left(t\right)=X_{L}\left(t_{j}\right)\;\mathrm{and}\;X_{G}\left(t\right)=X_{G}\left(t_{j}\right)\;\mathrm{for}\;t_{j}\leq t<t_{j+1}$$

This method, while simple, ensures the ODE solver has defined inputs. More advanced approaches, like NCDEs, could learn the continuous path $X\left(t\right)$ from discrete observations, offering a promising avenue for future refinement.

These continuous-time signals, $X_{L}\left(t\right)$ and $X_{G}\left(t\right)$, are then incorporated into the NODE dynamics function f (as defined in Equation (1)), ensuring the rate of change in the latent state is continuously influenced by the latest available guidance. The neural network f learns to interpret these signals and modulate the state evolution dH/dt accordingly, allowing the model to track conditions between sparser, high-accuracy corrections.

#### 3.4.2. Strong Correction

While continuous guidance helps, interval sensor (AVI) data $D_{A}$ provides sparse but highly accurate measurements, $\tau^{\mathrm{AVI}}$. The strong correction mechanism explicitly leverages this accuracy to counteract potential drift in $H\left(t\right)$. It functions as a discrete, event-triggered update.

We treat each valid AVI measurement for segment $s_{n}$ arriving at $t_{k}^{\mathrm{AVI}}$ as an event. The continuous integration (Equation (1)) pauses, and $H\left(t\right)$ is corrected. The challenge is that $\tau_{k}^{\mathrm{AVI}}$ is an Arrival-based Travel Time (ATT) over a past interval $\left[t_{\mathrm{entry}},t_{k}^{\mathrm{AVI}}\right]$. To handle this, we calculate a model-simulated ATT (${\hat{\tau }}_{\mathrm{ATT}}$) by integrating model-derived instantaneous speeds ${\hat{v}}_{n}\left(H\left(s\right);\psi_{n}^{\prime }\right)$ over the past interval:

(7) $${\hat{\tau }}_{k,n}^{\mathrm{AVI}}=\int_{t_{\mathrm{entry}}}^{t_{k}^{\mathrm{AVI}}}\frac{L_{n}}{{\hat{v}}_{n}\left(H\left(s\right);\psi_{n}^{\prime }\right)}ds$$

Here, $L_{n}$ is the segment length, and ${\hat{v}}_{n}\left(H\left(s\right);\psi_{n}^{\prime }\right)$ is derived from $H\left(s\right)$ via a learned function (parameterized by $\psi_{n}^{\prime }$). This requires accessing historical $H\left(s\right)$. The innovation ($e_{k}$) is the difference:

(8) $$e_{k}=\tau_{k}^{\mathrm{AVI}}-{\hat{\tau }}_{k,n}^{\mathrm{AVI}}$$

We denote $H\left(t_{k}^{\mathrm{AVI}-}\right)$ as the state before correction. The state is updated using:

(9) $$H\left(t_{k}^{\mathrm{AVI}+}\right)=H\left(t_{k}^{\mathrm{AVI}-}\right)+K_{k}e_{k}$$

where $H\left(t_{k}^{\mathrm{AVI}+}\right)$ is the corrected state, and $K_{k}$ ∈ $R^{d}$ is the adaptive gain vector, calculated as:

(10) $$K_{k}=P_{k}^{-}H_{k}^{T}{\left(H_{k}P_{k}^{-}H_{k}^{T}+R_{k}\right)}^{-1}$$

Here, $P_{k}^{-}$ ∈ $R^{d\times d}$ is the predicted state error covariance (model uncertainty), $R_{k}$ ∈ $R^{𝟙\times 𝟙}$ is the measurement noise covariance (observation uncertainty), and $H_{k}$ ∈ $R^{𝟙\times d}$ is the linearized observation operator (Jacobian matrix):

(11) $$H_{k}={\left.\frac{\partial {\hat{\tau }}_{\mathrm{ATT}}}{\partial H}\right|}_{H\left(t_{k}^{\mathrm{AVI}-}\right)}$$

This ensures $K_{k}$ adapts based on relative uncertainties. The corrected state $H\left(t_{k}^{\mathrm{AVI}+}\right)$ then serves as the new initial condition for the ODE solver, resuming integration from $t_{k}^{\mathrm{AVI}}$ using Equation (1). This mechanism stabilizes the model by anchoring it to high-confidence AVI data.

### 3.5. Model Training

The training process for FusionODE-TT involves optimizing the parameters of the neural networks involved: θ (dynamics function f), φ (decoder g), $\psi_{n}$ (observation functions $h_{n}$), and potentially any learnable parameters within the gain $K_{k}$. This optimization is achieved by minimizing a composite loss function using gradient-based methods and backpropagation through the entire computational graph, including the ODE solver and the discrete correction steps.

#### 3.5.1. Loss Function Design

The first type of loss is primary prediction loss ($L_{pred}$): This term directly measures the discrepancy between the model’s final predicted travel time ${\hat{\tau }}_{P}\left(t,\Delta t\right)$ for path p and the corresponding ground truth travel time $\tau_{P}^{true}\left(t,\Delta t\right).$ Suitable ground truth might come from held-out high-quality AVI data for the entire path or other reliable sources. Common choices include mean absolute error (MAE, *q* = 1) or mean squared error (MSE, *q* = 2).

(12) $$L_{\mathrm{pred}}=\frac{1}{N}\sum_{i=1}^{N}|{\hat{\tau }}_{p,i}\left(t_{i},\Delta t\right)-\tau_{p,i}^{\mathrm{true}}\left(t_{i},\Delta t\right){|}^{q}$$

where N is the number of samples in a batch, and i indexes the samples.

The second type is auxiliary AVI consistency loss ($L_{\mathrm{AVI}}$). To ensure that the latent state $H\left(t\right)$ evolves meaningfully and that the observation functions $h_{n}$ provide accurate segment estimates even before the strong correction is applied, we introduce an auxiliary loss term. This term penalizes the difference between the model’s segment travel time estimate immediately prior to correction $,h_{n}\left(H{\left(t_{k}^{AVI}\right)}^{-};\psi_{n}\right),$ and the actual observed AVI travel time $\tau_{k}^{AVI}$:

(13) $$L_{\mathrm{AVI}}=\frac{1}{M}\sum_{k=1}^{M}{\left|{\hat{\tau }}_{k,n_{k}}^{\mathrm{AVI}}-\tau_{k}^{\mathrm{AVI}}\right|}^{q}$$

where M is the number of AVI correction events occurring within the training batch/sequence, and $n_{k}$ denotes the segment associated with the *k*-th event. This loss encourages the continuous dynamics and the observation functions to remain consistent with the high-accuracy AVI data throughout the evolution.

The third type is guidance reconstruction loss ($L_{guide}$). In some cases, it might be beneficial to ensure that the latent state $H\left(t\right)$ retains sufficient information about the guidance inputs $\left(X_{L},X_{G}\right)$. If a reconstruction mapping $p\left(H\left(t\right)\right)$ back to the space of guidance features can be defined (e.g., another neural network), a loss term can be added:

(14) $$L_{\mathrm{guide}}=\frac{1}{T_{\mathrm{guide}}}\sum_{j}{\left|p_{\mathrm{rec}}\left(H\left(t_{j}\right)\right)-\left[X_{L}\left(t_{j}\right),X_{G}\left(t_{j}\right)\right]\right|}^{q}$$

where the sum is over relevant time steps $t_{j}$ where guidance data $X_{G}\left(t_{j}\right)$ and $X_{L}\left(t_{j}\right)$ are available. However, defining and training *q* adds complexity, and this term might not always be necessary if $L_{\mathrm{pred}}$ and $L_{\mathrm{AVI}}$ provide sufficient supervision.

Therefore, the total loss can be expressed as:

(15) $$L=L_{\mathrm{pred}}+\lambda_{1}L_{\mathrm{AVI}}+\lambda_{2}L_{\mathrm{guide}}$$

where $\lambda_{1}\geq 0$ and $\lambda_{2}\geq 0$ are hyperparameters that control the relative importance of the auxiliary loss terms.

#### 3.5.2. Optimization and Gradient Computation

The model parameters ($\theta ,\varphi ,\psi_{n}$, learnable parts of $K_{k}$) are optimized using stochastic gradient descent variants. The core challenge lies in computing the gradients of the loss L with respect to these parameters, as this requires backpropagation through the dynamics defined by the ODE solver and the discrete correction steps.

The standard technique for computing gradients through the ODE integration (Equation (4)) is the adjoint sensitivity method. This method avoids storing intermediate activation by solving a second, augmented ODE backward in time, offering constant memory complexity with respect to the number of integration steps. However, it can be computationally intensive and may suffer from numerical instability.

The strong correction step (Equation (9)) introduces discontinuities in the state trajectory $H\left(t\right)$. Backpropagating gradients through these discrete jumps requires careful handling. The gradient of the loss L with respect to the state after the correction, $\frac{\partial L}{\partial H\left(t_{k}^{\mathrm{AVI}+}\right)}$, needs to be correctly propagated to the state before the correction, $\frac{\partial L}{\partial H\left(t_{k}^{\mathrm{AVI}-}\right)}$, and to any learnable parameters in $K_{k}$ and those involved in calculating $e_{k}$ (i.e., $\psi_{n}^{\prime }$). If $K_{k}$ is learnable or depends on $H\left(t_{k}^{\mathrm{AVI}-}\right)$, its derivative path must also be considered. The relationship for propagating gradients across the update step (Equation (9)), assuming $K_{k}$ might depend on $H\left(t_{k}^{\mathrm{AVI}-}\right)$, can be derived using the chain rule. For instance, the gradient with respect to $H\left(t_{k}^{\mathrm{AVI}-}\right)$ would be:

(16) $$\frac{\partial L}{\partial H\left(t_{k}^{\mathrm{AVI}-}\right)}=\frac{\partial L}{\partial H\left(t_{k}^{\mathrm{AVI}+}\right)}\left(I+\frac{\partial K_{k}}{\partial H\left(t_{k}^{\mathrm{AVI}-}\right)}e_{k}+K_{k}\frac{\partial e_{k}}{\partial H\left(t_{k}^{\mathrm{AVI}-}\right)}\right)$$

where I is the identity matrix. Computing $\frac{\partial e_{k}}{\partial H\left(t_{k}^{\mathrm{AVI}-}\right)}$ involves differentiating Equation (8) with respect to $H\left(t_{k}^{\mathrm{AVI}-}\right)$, which in turn requires differentiating Equation (7) (the simulated ATT). The gradients with respect to parameters of $K_{k}$ (if any) and $\psi_{n}^{\prime }$ (via $e_{k}$) also need to be carefully derived and included in the backpropagation pass. The adjoint state must be appropriately updated or reset at these event times during the backward pass.

#### 3.5.3. Robustness of the Guided Fusion Mechanism

A critical prerequisite for the effective implementation of the guided fusion mechanism is ensuring the quality and validity of the AVI data that underpins it. To mitigate the risk of performance degradation from erroneous sensor readings, we employ a robust filtering methodology for all incoming AVI travel time observations, as proposed in [4]. This advanced filter leverages both within-day and day-to-day variations in traffic conditions to effectively identify and remove outliers caused by systematic errors or transient sensor failures, even in low-sample-size conditions.

Furthermore, the strong correction mechanism is designed to be resilient to data outages. In a scenario where a sensor failure results in a complete lack of validated AVI events within a given prediction horizon, the guidance loss term ($L_{\mathrm{AVI}}$) is not computed. In such cases, the model seamlessly relies on the uncorrected ODE trajectory derived from the historical data, ensuring the framework’s robustness and preventing performance degradation.

## 4. Results

### 4.1. Experimental Setup

The experiments are conducted on a selected path in Hong Kong. The study path is 17.8 km long and connects Tuen Mun New Town and Tsuen Wan New Town in New Territories, Hong Kong. The free-flow travel time is 14.3 min. The expressway selected for this study serves as a representative example of a complex urban corridor. Table 1 provides the traffic characteristics of the study path. It is seen in Table 1 that the path features multiple interchanges with significant on- and off-ramp traffic, weaving sections and bus stops. Its traffic dynamics are influenced by a mixture of commuter and commercial vehicle flow. By validating our model in such a challenging and dynamic environment, we aim to provide a strong baseline for its performance and applicability to other complex urban roads.

**Figure 2:**
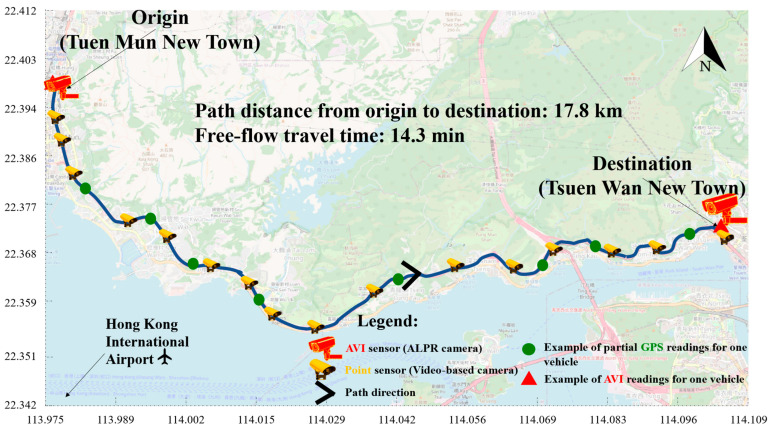
Map of study path.

Figure 2 gives the map of the study path. There are a pair of AVI sensors installed at both ends of the study path. Sixteen video-based cameras are located along the study path. Moreover, GPS data can be obtained from partial goods vehicles from commercial companies. Therefore, there are three types of traffic data that can be used for predicting travel times on the study path. The current advanced traveler information system, Speed Map Panel, also monitors the study path. It provides the path travel time estimates of the study path every 2 min. As the estimates have been validated with a field test [32], they are regarded as ground truth in this study. The invalid AVI data have been filtered out using the previous filtering algorithm in [4]. Data from 21 weekdays in May 2018 are used in the experiments. The last five weekdays are used for validation, and the remaining data are trained for the proposed FusionODE-TT model. The prediction horizon is set as 30 min ahead.

The performance of the proposed FusionODE-TT model was benchmarked against other deep learning models. The MAE and the mean absolute percentage error (MAPE) are used to evaluate the prediction performance, as shown in Equations (17) and (18).

(17) $$MAE=\frac{1}{T}\sum_{t_{0}=1}^{T}\left|{\hat{\tau }}_{p}\left(t_{0},∆t\right)-\tau_{p}^{\mathrm{true}}\left(t_{0},∆t\right)\right|$$

(18) $$MAPE=\frac{1}{T}\sum_{t_{0}=1}^{T}\frac{\left|{\hat{\tau }}_{p}\left(t_{0},∆t\right)-\tau_{p}^{\mathrm{true}}\left(t_{0},∆t\right)\right|}{\tau_{p}^{\mathrm{true}}\left(t_{0},∆t\right)}$$

where ${\hat{\tau }}_{p}\left(t_{0},∆t\right)$ are predicted values and $\tau_{p}^{\mathrm{true}}\left(t_{0},∆t\right)$ are true values in the validation set.

### 4.2. Performance Evaluation

Table 2 gives the overall performance of predicted path travel times using different sources of traffic data. It is found that the usage of three data sources can achieve the best prediction performance (MAPE = 3.1% and MAE = 0.46 min). Therefore, the rest of the experiments adopt all three data sources for TTP on the study path. Moreover, it is found that AVI-based predictions outperform the other data sources and combinations. This indicates that AVI is the most information-rich data source in this study.

The recent machine learning methods are selected for benchmark comparison. They are the LSTM model [33], the Gaussian Mixture Model (GMM) [20], and the Graph Convolutional Network (GCN) [34]. In recent years, these machine learning methods have shown excellent performance on travel time estimation and prediction problems and have therefore received widespread attention. Moreover, they are benchmarked in related studies [20,35]. Table 3 provides a comparison of these machine learning methods. It is observed that the proposed FusionODE-TT model outperforms the other prediction models (MAPE = 3.1% and MAE = 0.46 min).

**Figure 3:**
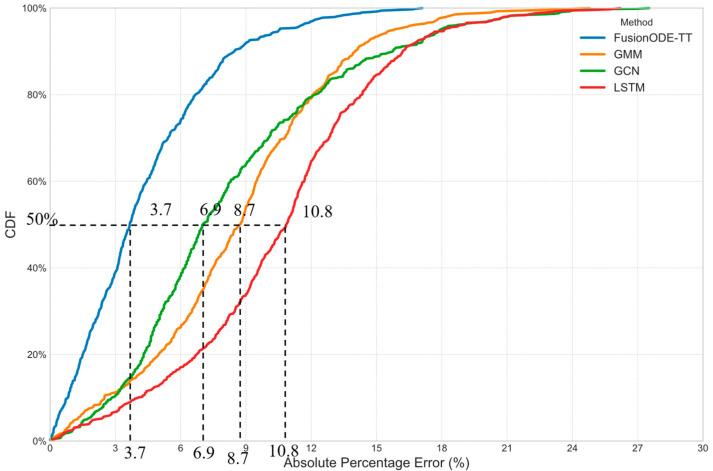
CDF of errors of predicted path travel times from different methods.

Figure 3 shows the cumulative distribution function (CDF) plots of the absolute percentage errors. It is seen that the proposed FusionODE-TT model outperforms the other methods. The medians of CDF vary from 3.7% to 10.8%. The largest difference between the proposed FusionODE-TT model and other methods is 7.1% (10.8% − 3.7% = 7.1%).

The robustness to data sparsity is verified in the following experiment. Different percentages of AVI, GPS, and point sensor data are randomly removed in the training process. The prediction performance of four methods is compared and shown in Table 4. There are two observations from Table 4. First, it is found that the robustness is satisfactory, as the MAPE is still less than 10% when 60% of the data is left for each data source. Second, the prediction accuracy is more sensitive to the amount of AVI data. When 20% of the data is removed for each data source, the corresponding MAPE is 4.8%. However, when 20% more AVI data is removed (from 80% to 60%), the MAPE has a 79% increment from 4.8% to 8.6%.

A key insight from this analysis is revealed when comparing these results to the performance of single-source models. For instance, the proposed model operating with only 60% of the available data from all three sensors achieves a MAPE of 9.5% and an MAE of 1.49 min. Critically, this level of performance is superior to that of the model trained on 100% of the AVI data alone (MAPE of 10.2%, MAE of 1.59 min). This finding demonstrates a core strength of the proposed fusion framework: it is more effective to fuse multiple, sparse, and partially incomplete data sources than to rely on a single, albeit complete, data source. This confirms the model’s enhanced applicability and robustness for real-world scenarios, particularly in areas where sensor infrastructure may be limited or suffer from intermittent data loss.

This analysis was performed on the proposed FusionODE-TT model as well as all baseline models (LSTM, ARIMA, and GMM). The results are presented in Table 4. The findings clearly demonstrate the superior robustness of the proposed FusionODE-TT framework. While all models exhibit some performance degradation as training data becomes more limited, the proposed model’s decline is significantly more graceful. When the training data is reduced to just 60% of its original volume, the MAPE of the FusionODE-TT model is still less than 10%. In stark contrast, the baseline models show much greater sensitivity to data sparsity: under the same conditions, the MAPEs for the LSTM, GMM, and GCN are 30.5%, 25.6%, and 19%, respectively. This result strongly suggests that the proposed model’s architecture, particularly its ability to fuse information from heterogeneous sources, provides a significant advantage in maintaining prediction accuracy in data-poor environments, which are common in real-world transportation systems.

To evaluate the effect of each component of the proposed FusionODE-TT model on prediction accuracy, we conduct a comprehensive ablation study. The study was designed to quantify the performance contribution of two core innovations: (1) the use of continuous-time dynamics modeled by the NODE shown in Section 3.3, and (2) the guided fusion mechanism using strong correction as illustrated in Section 3.4. We designed three experimental setups, which were evaluated on the same 30 min-ahead prediction task. Table 5 gives the performance of each component.

It is observed in Table 5 that both components are essential in the proposed FusionODE-TT model. First, the MAPE increases from 3.1% to 12.4% without ODE, which is a 300% increment. This suggests that modeling the underlying traffic dynamics as a continuous process, as enabled by the NODE, is better suited to capture the complex, non-linear evolution of traffic states compared to conventional discrete-step recurrent networks. Second, the MAPE increases 4.6% (7.7% − 3.1% = 4.6%) without the designed strong correction mechanism. This demonstrates that it is a highly effective strategy to leverage sparse but high-accuracy AVI data to guide and correct the continuous latent state. It helps the model anchor its understanding of the true traffic state, preventing the ODE from drifting over long prediction horizons and leading to more accurate and robust predictions.

Moreover, the results clearly demonstrate a trade-off between model complexity, accuracy, and computational efficiency in Table 5. The full FusionODE-TT model has an average inference time of 8.5 milliseconds per prediction. This is significantly faster than the 2 min temporal resolution of our prediction task, confirming that the model is highly suitable for real-time deployment in practical ITS.

Furthermore, the ablation study provides insight into this trade-off. The model without ODE, being the simplest architecture, is the fastest, with an inference time of just 3.2 milliseconds. However, this speed comes at the cost of a significant reduction in accuracy (a 308% increase in MAE compared to the full model). Conversely, removing the guided fusion mechanism provides only a marginal speed-up, yet still results in a noticeable drop in performance (a 178% increase in MAE compared to the full model). This analysis validates our design choices, demonstrating that the additional computational complexity introduced by the ODE and the guided fusion mechanism is a worthwhile investment, yielding substantial improvements in prediction accuracy while maintaining practical inference speeds for real-time applications.

**Figure 4:**
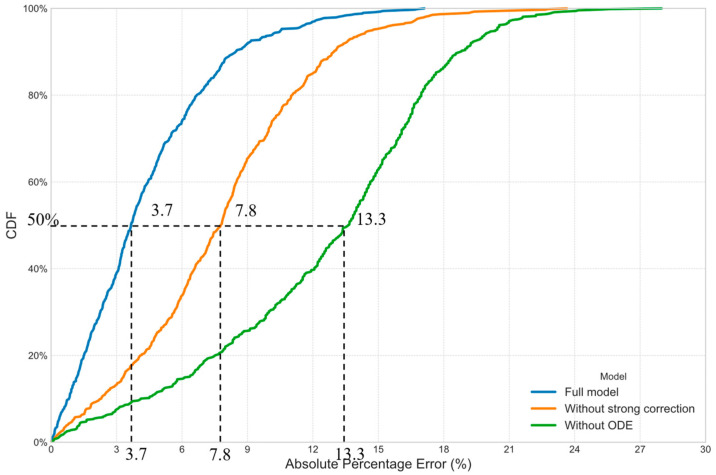
CDF of errors of predicted path travel times in the ablation study.

The CDF plots of absolute percentage errors in the ablation study are given in Figure 4. It is observed in Figure 4 that their distributions are significantly different. The medians of their distributions are 3.7%, 7.8%, and 13.3% for the full model, without strong correction, and without ODE, respectively. The ODE component is more essential to the performance compared with the strong correction mechanism.

**Figure 5:**
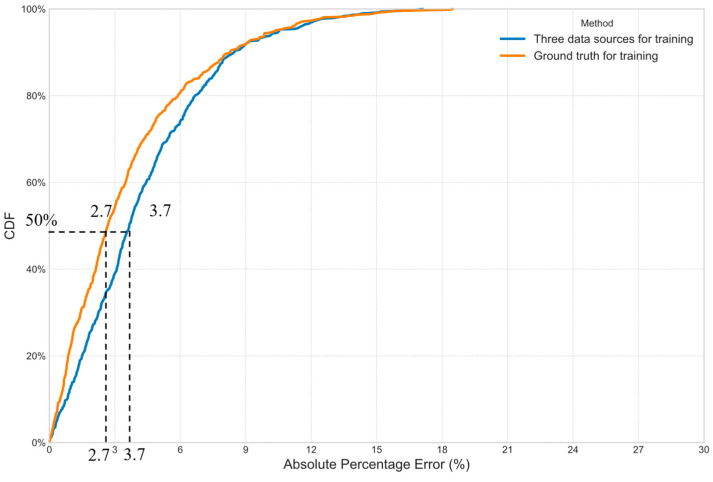
CDF of errors of predicted path travel times using three data sources and ground truth for training.

The proposed FusionODE-TT model fuses three sources of traffic data. It is worthwhile to compare the performance with the one using ground truth for training. It is noted that strong correction is removed for the case when the ground truth is used for training. It is because they require much less information from AVI data for correction. Figure 5 gives the comparison of the CDF of absolute percentage errors. It is seen in Figure 5 that the performance of the proposed FusionODE-TT model is satisfactory. The difference between the medians of CDF is only 1% (3.7% − 2.7% = 1%). It indicates that three data sources are properly fused for predicting path travel times.

## 5. Discussion

The experimental results presented in the previous section confirm the effectiveness of the proposed FusionODE-TT model. This section provides a broader discussion of the implications of these findings, the interpretability of the model, and the limitations of the current study, which in turn suggest directions for future research.

### 5.1. The Efficacy of Continuous-Time Guided Fusion

The superior performance of the full FusionODE-TT model, as demonstrated in the ablation study (Table 4), highlights the value of our two primary methodological contributions. First, the outperformance of the ODE-based models over the standard GRU baseline suggests that modeling traffic dynamics as a continuous-time process is a more effective approach. This allows the model to capture the underlying physical flow of traffic more faithfully than discrete-time models.

Second, and more critically, the significant performance gap between the “Guided” and “No-Guided” versions of the FusionODE-TT model validates the efficacy of the proposed guided fusion mechanism. This finding indicates that using sparse, high-fidelity AVI data to actively correct the model’s latent state trajectory is a powerful strategy. It effectively mitigates the problem of error accumulation and trajectory drift in long-term forecasting, demonstrating that the quality and strategic use of data can be more impactful than quantity alone.

### 5.2. Model Interpretability

While deep learning models offer powerful predictive capabilities, their “black box” nature can be a barrier to adoption in critical systems. Therefore, it is important to provide an interpretation of the internal state and mechanisms of the proposed FusionODE-TT model. We conducted a case study on a representative period from the test dataset that includes the morning peak transition (06:00–10:00).

**Figure 6:**
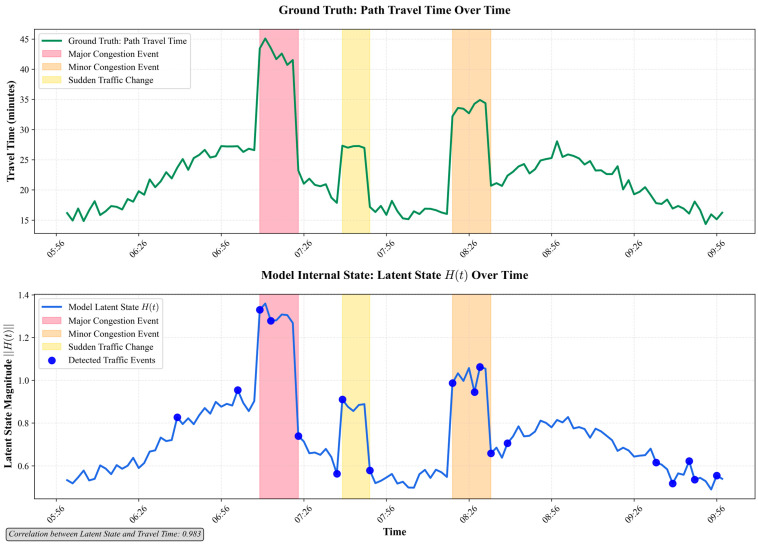
Interpretation on the model interval state $H\left(t\right)$.

Figure 6 shows the relationship between the magnitude of the model’s latent state $H\left(t\right)$ and the ground truth travel time. The model’s latent state (blue line) shows a clear correlation with the ground truth travel time (green line); its value spikes at the onset of congestion (e.g., around 07:00 and 07:30). The model is also able to automatically highlight periods of high volatility as detected traffic events (blue dots), demonstrating that the latent state has successfully learned to represent the intensity of the traffic state in a meaningful way. Furthermore, the coefficient of correlation between latent state and travel time is 0.983. It verifies the effectiveness of the internal latent state for capturing the variations in path travel times.

**Figure 7:**
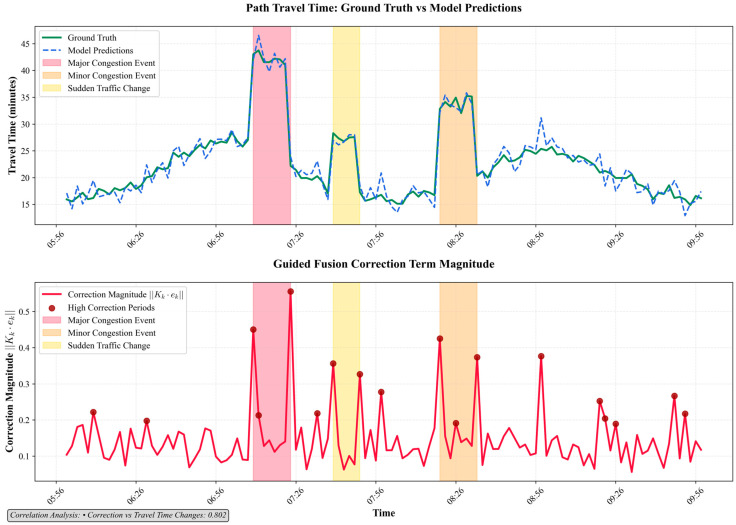
Interpretation on the strong correction.

Furthermore, we analyzed the magnitude of the correction term, $\left\|K_{k}\cdot e_{k}\right\|$, to verify that the guided fusion mechanism behaves in a logical and interpretable manner. As shown in Figure 7, the relationship between travel time change (defined as the difference in ground truth travel time between consecutive 2 min intervals) and the applied corrections is evident. During periods of stable traffic, such as before 06:30, the change in ground truth travel time between adjacent intervals is minimal, and consequently, the correction term magnitude (red spikes) remains close to zero.

However, as the morning congestion begins to build between 07:00 and 08:30, the traffic dynamics become more volatile. It is precisely during these periods that the guided fusion mechanism applies the strongest corrections, as indicated by the prominent red spikes. To quantify this relationship, we calculated the correlation between the correction term magnitude and the travel time change. The analysis yields a strong positive coefficient of correlation of 0.802, confirming that the model has learned to apply the most significant corrections when the real-world traffic state is changing most rapidly. This demonstrates that the guided fusion mechanism is not applying corrections arbitrarily but has learned an intelligent, responsive, and trustworthy strategy, which is critical for real-world deployment.

### 5.3. Limitations and Future Work

While this study provides strong evidence for the potential of the FusionODE-TT framework, we acknowledge several limitations that open avenues for future research. First, regarding real-time applicability, our computational analysis shows that the model’s inference time is well within the budget for typical traffic management cycles. However, the complexity of the Neural ODE presents a trade-off between accuracy and speed. Future work could explore model optimization techniques, such as quantization or pruning, to further enhance its efficiency for edge computing applications. Second, in terms of generalizability, the model was validated on a single, albeit complex, urban corridor. Future studies should test the framework’s transferability to cities with different road network topologies (e.g., grid-like vs. radial) to fully assess its robustness. Finally, the current model relies exclusively on traffic-related data. A significant opportunity for enhancement lies in the integration of external factors. Incorporating contextual data such as adverse weather conditions, traffic incidents, and special events as features could provide the model with a more comprehensive understanding of the factors influencing travel time, likely leading to further improvements in prediction accuracy.

## 6. Conclusions

This paper introduced FusionODE-TT, a novel model designed to address the persistent challenges of path travel time prediction from sparse and heterogeneous traffic data. By conceptualizing traffic dynamics as a continuous-time process, our model utilizes a NODE to capture the complex, non-linear evolution of the traffic state. The central contribution of our work is a guided fusion mechanism, which integrates high-fidelity but sparse AVI data to actively correct and anchor the model’s latent state trajectory.

The comprehensive experiments yield several key outcomes that validate the proposed approach. The quantitative results demonstrate that: (1) fusing data from all available sensors (AVI, GPS, point sensors) using the proposed model yields a significantly more accurate prediction (MAPE of 3.1%) than using any single data source, confirming the value of a holistic, heterogeneous fusion strategy (from Table 2); (2) the proposed model is significantly more robust to data scarcity than traditional baselines, with its error still less than 10% when training data is reduced to 60%, compared to nearly 20% for other methods (from Table 4); and (3) an ablation study proves that both the continuous-time dynamics (NODE) and the guided fusion mechanism are essential components, each providing a significant and independent contribution to the model’s superior performance (Table 5). Critically, our qualitative analysis further reveals that the model is not a “black box.” The model’s internal latent state is shown to be highly correlated with real-world traffic conditions (coefficient of correlation of 0.983), and the guided fusion mechanism behaves logically by applying the strongest corrections during periods of high traffic volatility (from Figure 6 and Figure 7).

Future research should focus on several promising directions. First, the guided fusion mechanism, while proven effective, could be enhanced by incorporating more sophisticated attention mechanisms to dynamically weight the influence of different AVI correction events based on their reliability or the current traffic state. Second, the current model architecture could be extended to a full spatiotemporal graph neural network, allowing it to model network-wide traffic propagation more effectively. Furthermore, the current framework relies exclusively on traffic-related sensor data. To create a more comprehensive prediction system, a significant avenue for future work is the integration of external contextual factors. Variables such as adverse weather conditions (e.g., rainfall intensity), traffic incidents, and special events could be incorporated as additional features into the input sequence. This would allow the model to learn the complex, non-linear impacts of these external events on traffic dynamics, further improving its prediction accuracy and practical utility for advanced traffic management.

## Figures and Tables

**Table 1:** Traffic characteristics of the study path.

Characteristic	Value
Road type	Expressway
Path distance (km)	17.8
Number of bus stops	20
Number of entries along the study path	17
Number of exits along the study path	19
Free-flow travel time (min)	14.3
Speed limits (km/h)	70 (47%), 80 (53%)

**Table 2:** Overall performance of predicted path travel times using different sources of traffic data.

Data Source	MAPE (%)	MAE (min)
A	10.2	1.59
G	15.2	2.23
P	12.8	1.91
A + G	4.9	0.73
A + P	3.5	0.52
P + G	4.8	0.71
A + G + P	3.1	0.46

A: AVI sensors; P: point sensors; G: GPS sensors.

**Table 3:** Benchmark comparison of the overall performance of predicted path travel times.

Method	MAPE (%)	MAE (min)
FusionODE-TT	3.1	0.46
LSTM	10.3	1.61
GMM	8.8	1.39
GCN	6.4	0.98

**Table 4:** Effects of the quantity of data used in training on the overall performance of predicted path travel times.

Percentage of Data Left (%)			FusionODE-TT		LSTM		GMM		GCN	
AVI Data	GPS Data	Point Sensor Data	MAPE (%)	MAE (min)	MAPE (%)	MAE (min)	MAPE (%)	MAE (min)	MAPE (%)	MAE (min)
100	100	100	3.1	0.46	10.3	1.61	8.8	1.39	6.4	0.98
80	80	80	4.8	0.71	15.4	2.28	12.9	1.91	9.6	1.42
80	80	60	5.1	0.76	16.4	2.44	13.7	2.04	10.2	1.52
80	60	80	4.9	0.72	15.7	2.31	13.2	1.94	9.8	1.44
80	60	60	5.8	0.84	18.6	2.70	15.6	2.26	11.6	1.68
60	80	80	8.6	1.36	27.6	4.37	23.1	3.66	17.2	2.72
60	80	60	9.1	1.43	29.2	4.59	24.5	3.85	18.2	2.86
60	60	80	8.9	1.41	28.6	4.53	23.9	3.79	17.8	2.82
60	60	60	9.5	1.49	30.5	4.78	25.6	4.01	19.0	2.98

**Table 5:** Effects of each component in the proposed FusionODE-TT model on the overall performance of predicted path travel times.

Components	MAPE (%)	MAE (min)	Inference Time Per Prediction (ms)
Full model	3.1	0.46	8.5
Without ODE	12.4	1.88	3.2
Without strong correction	7.7	1.28	8.1

## Data Availability

Data are contained within the article.
